# Prophylactic and Therapeutic Breast Conservation in *BRCA1/2* Mutation Carriers

**DOI:** 10.4061/2011/481563

**Published:** 2011-09-04

**Authors:** Randal L. Croshaw, Megan L. Marshall, Tesha L. Williams, Kathleen M. Erb, Thomas B. Julian

**Affiliations:** ^1^Allegheny General Hospital, 320 E North Avenue, Pittsburgh, PA 15212-4746, USA; ^2^Drexel University College of Medicine, Allegheny Campus, Pittsburgh, PA 15212-4746, USA; ^3^National Surgical Adjuvant Breast and Bowel Project, Pittsburgh, PA 15212-5255, USA

## Abstract

Breast-conserving therapy (BCT) for sporadic breast cancer has been widely accepted by surgeons and patients alike. While BCT is associated with a higher risk of ipsilateral breast tumor recurrence (IBTR), it has not been shown to decrease overall survival (OS) in comparison with mastectomy. Many women with a *BRCA1/2* mutation opt for mastectomy instead of breast-conserving measures at the time of a breast cancer diagnosis. In some cases, this is due to fear of aggressive disease, but to date, there have been no studies offering strong evidence that breast conservation should not be offered to these women. *BRCA1/2*-associated breast cancer has not been found to be more aggressive or resistant to treatment than comparable sporadic tumors, and no study has shown an actual survival advantage for mastectomy in appropriately treated affected mutation carriers. This paper reviews the available literature for breast conservation and surgical decision making in *BRCA1/2* mutation carriers.

## 1. Introduction

Knowledge about *BRCA1* and *BRCA2* and how patients with mutations should be treated has been slow to develop since the discovery of these genes in 1990 and 1994, respectively [1, 2]. Many factors have limited collection of data on this subject, such as availability of testing, expense, fear of testing, and the small number of patients available for study. The resultant lack of knowledge drives and sustains patient anxiety, sometimes prompting them to select mastectomy in hopes of a cure while sacrificing cosmesis, body image, and perhaps sexuality [3, 4]. In a 2003 study by van Oostrom et al., 21 of 23 BRCA1/2 mutation carriers underwent prophylactic mastectomy [5]. These patients reported a less favorable body image, while 70% of them reported changes in their sexual relationships. Prophylactic mastectomy has been shown to reduce the risk of breast cancer incidence or recurrence, but there is insufficient data to support an improvement in survival in affected or unaffected carriers, as discussed later [6–8]. Management decisions in *BRCA1/2* carriers prophylactically or at the time of diagnosis of an invasive cancer are complex. Patients will put different weights on different aspects of treatment, some favoring reducing the anxiety associated with surveillance and testing and some favoring body image. There is insufficient evidence at this time to forgo surveillance and breast conservation as viable options for *BRCA1/2* mutation carriers, options offered to patients with most other forms of breast cancer.

## 2. Tumor Characteristics of *BRCA1/2*-Associated Breast Cancer

*BRCA1/2* mutation carriers are known to have an increased incidence of breast cancer and premalignant lesions [9–13]. Histological differences in *BRCA1*-associated tumors as compared to sporadic tumors have been well described. *BRCA2*-associated tumors differ from those of noncarriers to a lesser extent. Invasive ductal carcinoma, NOS (not otherwise specified) type, is the most common histologic form of all hereditary breast cancers, including *BRCA1- *and *BRCA2-*associated breast cancers [14]. Most *BRCA1*-associated tumors have the basal-like phenotype and are more frequently ER−, PR−, Her2−, grade III, and of the medullary subtype. The majority of studies show no difference in tumor size or nodal status, factors known to be more important for survival based on our current staging of breast cancer [15–26]. The basal-like phenotype is rarely found in *BRCA2 *breast cancers. *BRCA2 *breast carcinomas tend to be ER+, PR+, Her2−, and higher grade than sporadic age-matched controls [27]. The growth rate of *BRCA1/2*-associated breast tumors is not accelerated. Multivariate analysis revealed that the apparent faster rate of growth in *BRCA1/2*-associated tumors was found to be associated only with a younger age at diagnosis and premenopausal status [16, 28]. 

No study has shown that *BRCA1/2*-associated breast cancer is more resistant to chemotherapy than are sporadic breast cancer controls. This issue was specifically addressed in a paper by Robson et al. in 2004, who found that the addition of chemotherapy negated any survival difference in the *BRCA1* mutation carriers compared with sporadic controls [19]. Kriege et al. was similarly able to show that *BRCA2*-associated tumors were more sensitive to anthracyclines than sporadic tumors [29]. Both preclinical and clinical studies have found that *BRCA1*-associated breast tumors are sensitive to platinum agents and cyclophosphamide [30–34]. These studies are small but confirm an increased complete clinical response in *BRCA1/2-*associated cancers compared to sporadic cases. 

There was early concern that *BRCA1/2*-associated tumors were particularly radiosensitive [35], and because of the role of these genes in DNA repair [35–37], that radiation therapy may stimulate additional tumor formation. Studies by Gaffney et al. and Pierce et al. found no evidence that radiation therapy in this patient population is associated with increased complications, recurrence, or with increased tumorigenesis [24, 38].

Many women fear that *BRCA1/2*-associated cancers are more aggressive than are sporadic tumors. The most important question to answer here is whether there is a difference in breast cancer specific survival (BCSS) or overall survival (OS) between hereditary and sporadic tumors. Numerous studies have addressed this issue, but they are limited by small patient numbers and differences in treatment. To date, we have found no studies that show a statistically significant difference in BCSS or OS after a breast cancer diagnosis when *BRCA1/2* carriers are compared with matched sporadic controls [15, 18–21, 23, 25, 38–41].

## 3. Breast Conservation Therapy in Affected Mutation Carriers

There are limited studies evaluating breast conservation for *BRCA1/2* mutation carriers diagnosed with breast cancer (affected carriers). The information we seek to learn is in regard to the risk of ipsilateral breast tumor recurrence (IBTR), the risk of contralateral breast cancer (CBC), breast cancer-specific survival (BCSS), and OS. In general, the evidence available for evaluation is found in small retrospective studies, most with relatively short followup. The small number of patients and lack of long-term followup are important concerns, as it is the number of events that provides the needed information (Table 1). Ipsilateral or contralateral disease can be new or recurrent disease. In the ipsilateral breast, different quadrants or different histology can be indicators of new primaries. Contralateral tumors, invasive or noninvasive, are assumed to be new primaries. 

Several reports by Pierce et al. about breast conservation in *BRCA1/2* mutation carriers stand out, as they directly compare mastectomy to breast conserving therapy (BCT) [40]. Their latest study, published in 2010, compares breast conservation to mastectomy in 655 women with *BRCA1/2* mutations diagnosed with breast cancer. 302 of these women who underwent BCT and 353 who underwent unilateral mastectomy were followed for 8.2 years and 8.9 years, respectively. The estimated 15-year rate of IBTR was 23.5% in the BCT group and 5.5% in the mastectomy group (*P* = 0.0001, HR 4.5). The ipsilateral recurrences were felt to be second primaries in 16 of 23 (70%) cases. Two other studies published in the last 10 years also show a difference in IBTR between these two groups. Haffty et al. reported findings of 22 patients with a *BRCA1/2* mutation compared to 105 women without this mutation, all of whom underwent BCT [22]. All of the study participants were diagnosed with breast cancer by the age of 42 and were followed for a median of 12 years. These researchers reported an IBTR rate of 49% in the mutation carriers compared to 21% in the controls (*P* = 0.007). They similarly believed that these ipsilateral recurrences were second primaries rather than true recurrences. Garcia-Etienne et al. followed 54 *BRCA1/2* patients and 162 sporadic controls treated with BCT with a median followup of 4 years [23]. They reported a projected 10-year rate of IBTR of 27% in mutation carriers compared to 4% in the sporadic controls, HR 3.9, *P* = 0.03 based on 6 events in the carrier arm and 4 events in the control arm. Criticisms of these studies include the observations that there was a statistically significant difference in the mean age of the *BRCA1/2* mutation carriers (33 years) and the sporadic controls (37 years), (*P* = 0.001) and that none of the mutation carriers underwent bilateral salpingo-oophorectomy (BSO), nor did they receive hormonal therapy (as compared to the 15% in the sporadic group, *P* = 0.05) in the study by Haffty et al. [22]. Both studies by Pierce et al. and Garcia-Etienne et al. project their data over longer time points to estimate a difference in tumor recurrence [23, 40]. 

Several other informative studies have been published reporting a rate of IBTR in patients undergoing BCT that contradicts the findings reported above. An earlier study by Pierce et al. revealed that there was no difference in IBTR between 160 *BRCA1/2* mutation carriers and 445 matched controls all undergoing BCT with a median followup of 6.7 to 7.9 years [21]. Kirova et al. found no difference (*P* = 0.13) in IBTR when they compared 27 *BRCA1/2* mutation carriers to 104 patients with a family history of breast cancer to 261 matched controls with a 13.4 year followup [20]. Kirova at al. found age to be the most significant predictor of IBTR, with a relative risk (RR) of 1.05 for each decreasing year of age (*P* = 0.01). Two other studies have reported no difference in IBTR, but these differ in their methods. Brekelmans et al. reported no difference in IBTR in 226 patients with a family history of *BRCA1/2* compared with 311 patients with a family history of breast cancer but testing negative for *BRCA1/2* in the family, and compared to 759 patients with sporadic breast cancer, with a mean duration of followup from 4.3 to 5.1 years [25]. Robson et al. also found no difference in IBTR at 10 years when they compared 28 women of Ashkenazi descent with *BRCA1/2* mutations to 277 women of Ashkenazi descent without mutations, but they did find age <50 to be a statistically significant risk of IBTR on univariate analysis (16% versus 6%, *P* = 0.01) and on multivariate analysis (RR 2.51, *P* = 0.01) [41]. Interestingly, it appears that age at diagnosis rather than *BRCA1/2* mutation status is prognostically important in predicting IBTR [20, 22, 41]. 

With regard to the risk of contralateral breast cancer, Pierce et al. reported no significant difference in the rate of CBC between the 302 *BRCA1/2* mutation carriers who underwent BCT and the 353 *BRCA1/2* mutation carriers who underwent unilateral mastectomy [40]. All of the other studies we reviewed did reveal a difference in CBC ranging from 1% to 11% in controls to 25% to 42% in mutation carriers when *BRCA1/2* mutation carriers were compared to patients with familial, non-*BRCA1/2*-associated breast cancer and/or sporadic controls [20–23, 25, 41, 42]. 

We found no study that showed a survival difference when BCT was compared to mastectomy in *BRCA1/2* mutation carriers or when BCT was compared in *BRCA1/2* mutation carriers and familial, non-*BRCA1/2*-associated breast cancer and/or sporadic controls [19–21, 23, 25, 40, 41]. Survival is influenced by stage of disease at diagnosis, tumor characteristics, and treatment. Triple-negative tumors appear to influence survival; therefore, the mix of *BRCA1* and *BRCA2* cases in a given paper may also influence survival data. Because of the small patient population reported in these papers, we are not able to separately evaluate *BRCA1* and *BRCA2* for IBTR, CBC, BCSS, and OS in triple-negative breast cancer. Due to the increased risk of IBTR and the elevated risk of CBC in *BRCA1/2* mutation carriers, risk-reducing strategies such as BSO or the use of tamoxifen, for those with ER-positive tumors, should be employed as they have been shown to reduce breast cancer recurrence [43].

## 4. Risk Reduction with Bilateral Prophylactic Mastectomy (BPM), Bilateral Salpingo-Oophorectomy (BSO), and Tamoxifen in Unaffected Mutation Carriers

Many women elect to undergo bilateral prophylactic mastectomy (BPM) sometime after learning of their *BRCA1/2* mutation status. The reasons for this decision are complex and will be discussed in the next section. The benefits of BPM in unaffected mutation carriers are known primarily through the results of a few published studies. The Prose study group has published two papers that detail the benefits of BPM. Their first paper, published in 2004, followed 483 women with a *BRCA1/2* mutation for a mean of 6.4 years [6]. They reported a 1.9% prevalence of breast cancer in 105 women who underwent BPM compared with 48.7% of 378 women undergoing surveillance only. Women who had undergone prophylactic BSO were excluded. Their followup study with additional patients, reported in 2010 by Domchek et al. with three years of prospective followup, also noted a difference in breast cancer diagnosis, from 0% in the BPM group to 7% in the non-BPM group [44]. A study predating these, by Meijers-Heijboer et al. [7] prospectively followed 139 women with *BRCA1/2* mutations for a mean of 2.9 to 3 years [7]. 76 of these women underwent BPM and were found to have no breast cancers diagnosed, compared with 8 of 63 women (12.7%) who underwent screening only (*P* = 0.003). The authors do not provide data related to survival differences in these papers, as the number of events is too small, as is the length of followup [6, 7, 44].

The preventive benefit of prophylactic BSO does appear to reduce the prevalence of breast cancer as well as to improve BCSS and to improve OS in premenopausal *BRCA1/2* mutation carriers [25, 40, 44]. Therefore serious consideration should be given to this preventive measure in women after they complete their families, ideally between 35 and 40 years or individualized based on the earliest age of ovarian cancer diagnosis in the family. Women who are *BRCA1/2* mutation carriers and choose not to undergo prophylactic BSO may wish to consider the use of tamoxifen, as it has been shown to reduce breast cancer incidence in that group [43, 45]. For young women with *BRCA1/2* mutations or who are otherwise at high risk for breast cancer, the current American Cancer Society guidelines recommend the inclusion of screening MRI, which has been shown in several studies to have increased sensitivity (79.5% to 91%) for T2 or smaller breast cancer compared to mammography with or without the addition of ultrasound (33% to 50%) [46–49]. No recommendations exist for surveillance after a diagnosis of breast cancer in this population.

## 5. Surgical Decisions in Affected and Unaffected *BRCA1/2* Mutation Carriers

Several studies have addressed surgical decision making in unaffected *BRCA1* and *BRCA2* mutation carriers. The reported proportions of women who adopt risk-reducing bilateral mastectomy versus intense surveillance are highly variable. A limited review of the literature revealed six studies that analyzed the utilization of risk-reducing mastectomy versus surveillance in unaffected women identified as carrying a *BRCA1 *or *BRCA2 *gene mutation [50–55].

In a study of 279 adult male and female members of families with an identified *BRCA1 *gene mutation from a registry maintained by the Creighton University Hereditary Cancer Institute, 43% requested *BRCA1 *test results [50]. Of those tested for the previously identified familial mutation, 46% (53 of 115 electing to receive results) were mutation carriers. Among the unaffected *BRCA1 *mutation carriers with no previous prophylactic surgery, 17% (2 of 12) intended to have risk-reducing bilateral mastectomy, but none had actually undergone surgery 1 month after *BRCA *testing. Likewise, Botkin et al. documented a low rate of risk-reducing bilateral mastectomy among 37 unaffected *BRCA1/2 *mutation carriers [51]. At 2 years after testing, no women had undergone mastectomy for cancer prophylaxis; however, some reported that they were strongly considering surgery. Of those women, 2 of the 20 women in the younger (25–39 years) age group stated that they were considering this procedure, and 2 of the 12 women in the older (40 years and older) carrier group stated that they were considering it. In addition, Botkin et al. reported an increase in the utilization of mammography and self-breast examination among unaffected carrier females who did not chose surgery. 

In contrast, three studies demonstrated a larger number of unaffected *BRCA *carriers who elected to undergo BPM. In a Rotterdam-based study of unaffected women with an identified mutation who were eligible for prophylactic surgery, 51% (35 of 68 women) opted for risk-reducing bilateral mastectomy [52]. The authors reported that of these women, there was a tendency towards mastectomy at younger ages; most were between 30 and 44 years old. Also, the decision to undergo BPM was often made within the first year following receipt of their *BRCA* test results. Lodder et al. found that 53.8% of mutation carriers (14 of 26 unaffected *BRCA *carriers) underwent BPM one year after genetic testing [53]. They reported that often the decision for preventive surgery was made before the disclosure of test results for women who had a 50% *a priori* risk of carrying a known familial *BRCA *mutation. Lastly, in the largest prospective study of 251 individuals with confirmed *BRCA *mutations at Memorial Sloan-Kettering Cancer Center, 29 of 194 women (14.9%) who had breast tissue at risk at the time they received genetic test results underwent bilateral mastectomy for cancer prophylaxis within a median of 5.3 months after receiving results [54]. Twenty of the 233 *BRCA *carriers had previously elected BPM based on family history alone. Data presented at the Facing Our Risk of Cancer Empowered *Joining FORCEs *conference in 2007 by Dr. Steve Narod and published in 2008 by Metcalf et al. discussed international variation in the decision to undergo prophylactic surgeries [55, 56]. Narod introduced data from an ongoing study of 8,058 known *BRCA1 *or *BRCA2 *mutation carriers from 11 countries. Within a four-year followup period, 36% of unaffected carriers from the United States chose BPM. Overall 248 (18%) of the 1382 unaffected carriers from all countries chose prophylactic mastectomy. 

Many BRCA1/2 mutation carriers diagnosed with breast cancer (affected carriers) opt for bilateral mastectomy rather than BCT for initial treatment of disease. Therefore, hereditary cancer risk assessment at the time of diagnosis may significantly affect a woman's treatment decisions. Four studies investigating surgical decision making in *BRCA *carriers at breast cancer diagnosis were identified through our literature search. Weitzel et al. found that 7 of 32 (22%) women with a newly diagnosed breast cancer carried a deleterious *BRCA *mutation [57]. All 7 of these women opted for contralateral prophylactic mastectomy. In a prospective study of 194 newly diagnosed breast cancer patients at Lombardi Cancer Center, 31 women were identified as carrying a *BRCA *mutation [58]. Forty-eight percent of these carriers chose bilateral mastectomy as the definitive surgical treatment for their breast cancer. Evans et al. identified 20 of 70 newly diagnosed breast cancer patients younger than 50 as *BRCA *mutation carriers [59]. Four of these women were aware of their genetic status at the time of diagnosis and three elected bilateral mastectomy. Four women were told of their mutation status within four weeks of diagnosis. Of these, two opted for bilateral mastectomy although one chose delayed contralateral mastectomy. The remaining 12 mutation carriers were told of their genetic status 3–36 months after diagnosis. Of these 12 women, one opted for contralateral mastectomy. In the data presented by Narod, the percentage of breast cancer survivors choosing contralateral prophylactic mastectomy was outlined for 8 countries, with Israel showing the largest proportion of prophylactic surgery (52%) followed by the United States (49%), Canada (28%), France (20%), Austria (16%), Italy (6%), and Poland (4%) [55]. 

For women and families who are found to carry a variant of uncertain clinical significance (VUS) in either *BRCA1 *or *BRCA2*, screening recommendations are offered according to personal and family history of cancer. There are few reports documenting prophylactic surgery and screening behaviors of individuals whose *BRCA *test results revealed a VUS, which is most likely a reflection of medical management guidelines published by the National Comprehensive Cancer Network, the National Society of Genetic Counselors, and the American Society of Clinical Oncology [60–62]. Surgical decision making in the context of a *BRCA *VUS is also based on personal and family history, as a VUS is uninformative with respect to cancer risk. Those individuals with classic hereditary breast-ovarian cancer syndrome may elect risk-reducing surgery based on family history alone and their own level of concern and anxiety. There are no published data to support prophylactic surgery in the equivocal risk population based on a *BRCA *VUS. Weitzel et al. reported that one of three women identified with a VUS at the time of a breast cancer diagnosis elected risk-reducing mastectomy even though they were counseled that the results of the *BRCA* sequencing were uninformative [57]. Those patients cited fear of cancer and uncertainty associated with the finding of a VUS as reasons for pursuing mastectomy. 

Although the risk of breast cancer in other hereditary cancer predisposition syndromes, such as Li-Fraumeni and Cowden syndrome, is known to be significantly elevated above that in the general population (as high as 50%), the efficacy of appropriate medical management and cancer prevention options for these patients has not been widely studied; therefore, they are based mainly on expert opinion. Current recommendations suggest discussing the option of prophylactic mastectomy on a case-by-case basis, including a detailed discussion reviewing the risk reduction benefit, cancer risks, and available reconstruction options [60].

Many factors contribute to the decision-making process with respect to increased surveillance and prophylactic surgeries for mutation carriers. The process is complex and can have a strong psychological impact, a subject that has also been extensively explored in the literature. Unaffected *BRCA *carriers who elect prophylactic mastectomy often report a higher perceived risk of developing breast cancer, higher levels of cancer-related worry, and higher levels of cancer-related distress than those who opt for surveillance. Individuals choosing risk-reducing surgery may also be aware of the hereditary nature of cancer in the family for a longer period of time and report a greater number of relatives affected with breast and ovarian cancer in the family, resulting in more first-hand experience with cancer and making them more apt to consider surgery for risk reduction [53, 63, 64]. Many studies have shown that women who choose bilateral mastectomy tend to be younger (between 30 and 43 years of age), have young children, and have a fear of leaving behind young children [52, 53, 64]. Surgical risk reduction is also sought with the intent to prolong life, the relief associated with a significant reduction in cancer risk, the negative pathologic characteristics of the tumor, and the desire to avoid further surgery at a later stage of cancer [53, 54, 59]. 

In contrast, *BRCA *mutation carriers who opt for surveillance cite reasons relating to possible dissatisfaction with general body image following mastectomy, the sexual relationship, and their trust in surveillance modalities. Women who opt for surveillance report being worried that they will not feel feminine, will not feel sexually attractive, and will have problems in the intimate relationship following mastectomy [53]. Unaffected *BRCA *carriers opting for surveillance specifically say that prophylactic bilateral mastectomy is too drastic an intervention and that they are reluctant to have healthy breast tissue removed. In addition, these women feel that they have time to explore the options of surgical risk reduction while undergoing surveillance. However, it is recognized that these attitudes may change over time, especially after a longer period of intensive breast surveillance and failure to comply with followup recommendations [64].

The majority of women who elect risk-reducing mastectomy are satisfied with their decision [65]. Those women who do regret undergoing mastectomy report that the dissatisfaction stems from surgery complications, poor cosmetic outcome, residual pain, fear that reconstruction will impede breast cancer detection, poor self-image, sexual dysfunction, lack of psychological support after surgery, and the fact that the subject of mastectomy was initiated by the physician rather than by the patient herself [53, 65–67]. With regard to the latter, many studies have illustrated that physician recommendations are an important determinant of surgical decisions, especially for women with a newly diagnosed breast cancer [58].

The decision to undergo prophylactic mastectomy for both unaffected *BRCA *mutation carriers and newly diagnosed carriers is a major, irreversible decision. Although a minority of *BRCA *carriers choose prophylactic mastectomy over intense surveillance, the majority of women who choose mastectomy are satisfied with their decision, report reduced cancer-related anxiety about developing breast cancer following surgery, and report favorable psychosocial outcomes.

## 6. Conclusions

It is clear that women with *BRCA1/2* mutations have a much higher risk of developing breast cancer than the 12.5% lifetime risk of the general population [68]. The risk of developing breast cancer by the age of 70 for carriers of *BRCA1* is 57% to 65%, while the risk in *BRCA2* carriers is slightly lower, at 45% to 49%, based on the findings of two recent metaanalyses [12, 13]. Many women choose to undergo bilateral prophylactic mastectomy, which has been shown to reduce the incidence of breast cancer in these patients, after learning that they are mutation carriers [6–8, 44]. Reduced BCSS in unaffected carriers is assumed although it has not been objectively quantified because of the short duration of followup and low number of events in these studies. 

It appears that many women diagnosed with an invasive breast cancer associated with a *BRCA1/2* mutation or a strong family history choose mastectomy as their definitive surgical therapy in lieu of breast conservation with irradiation. Other authors have reported the increased use of mastectomy over breast conservation in the general population with sporadic breast cancers for a variety of reasons as well [69, 70]. It has been shown that affected carriers undergoing mastectomy rather than BCT have a lower incidence of IBTR [19–23, 25, 40, 41]. Perhaps there is the benefit of “peace of mind” that comes with the reduced IBTR that those treated with BCT, and therefore, still subject to screenings and biopsies, do not have.

It has not been established that mastectomy at the time of a cancer diagnosis is the best therapeutic option, as several studies have shown that the BCSS and/or the OS in *BRCA1/2* is no different than that of sporadic cancers [15, 18, 39, 71, 72]. Studies evaluating breast conservation for the treatment of invasive disease in *BRCA1/2* carriers compared to that in sporadic controls reveal mixed results related to IBTR, but none have shown a difference in BCSS and/or OS [20, 22, 23, 25, 41, 71]. One study that compared mastectomy to breast conservation in *BRCA1/2* carriers noted an increased IBTR but no difference in OS [40]. For *BRCA1/2* mutation carriers who opt for BCT, risk-reducing strategies, such as the use of tamoxifen (for ER+ tumors) or BSO, are appropriate, as they appear to reduce the risk of IBTR and CBC [21, 22, 40, 43, 45]. No individual recommendations can be made for *BRCA1* apart from *BRCA2* mutation carriers, as most studies consider them together and have thus far had inadequate numbers to segregate the two despite their histologic differences and the proposed differences in cell function that* BRCA1* and *BRCA2* control [73].

At this time, the use of breast-conserving therapy in patients who are mutation carriers or who have a very strong family history for breast cancer should be handled on a case-by-case basis. Patients should be evaluated and informed about the current existing data and outcomes before an ultimate surgical decision is made. Consultation with a genetic counselor may be of benefit in helping the patient make an informed decision.

## Figures and Tables

**Table 1:** Outcomes of affected BRCA1/2 mutation carriers.

Study	Design	Patients	Followup	IBTR	BCSS	OS
Pierce et al. [40]	1	BCT = 302 Mast. = 353	8.2 to 8.9 years. Data projected to 15 years	BCT = 23.5% Mast. = 5.5%	BCT = 91.7% Mast. = 92.8% *P* = 0.85	BCT = 87.3% Mast. = 89.8% *P* = 0.73

Haffty et al. [22]	2	BRCA = 22 Sporadic = 105	12.7 years	BRCA = 41% Sporadic = 19% *P* = 0.007		

Garcia-Etiene et al. [23]	3	BRCA = 54 Sporadic = 162	4 years. Data projected to 10 years	BRCA = 27% Sporadic = 4% *P* = 0.03		

Pierce et al. [21]	4	BRCA = 160 Sporadic = 445	6.7 to 7.9 years. Data projected to 15 years	BRCA = 24% Sporadic = 17% *P* = 0.19		

Kirova et al. [20]	5	BRCA = 27 Familial = 104 Sporadic = 261	13.4 years	BRCA = 45% Familial = 31% Sporadic = 24% *P* = 0.33		Not significant at 20 years. Actual rates not reported.

Brekelmans et al. [25]	6	BRCA = 326 Familial = 311 Sporadic = 759	4.3 to 5.1 years. Data projected to 10 years	BRCA = 20 to 25% Familial = 6% Sporadic = 5% *P* = 0.001	BRCA = 62 to 68% Familial = 70% Sporadic = 59% *P* = 0.17	BRCA = 50 to 60% Familial = 66% Sporadic = 55% *P* = 0.32

Robson et al. [41]	7	BRCA = 28 Sporadic = 277	10.3 years	BRCA = 22% Sporadic = 7% *P* = 0.25	BRCA = 72% Sporadic = 87% *P* = 0.02*	BRCA = 66% Sporadic = 81% *P* = 0.05*

Robson et al. [19]	8	BRCA = 56 Sporadic = 440	9.7 years		BRCA1 = 63% BRCA2 = 86% Sporadic = 86% *P* = < 0.0001**	

Abbreviations: IBTR: in-breast tumor recurrence; BCSS: breast cancer-specific survival; OS: overall survival; BCT: breast conserving therapy; Mast.: mastectomy; BRCA: *BRCA1/2* unless otherwise specified.

Study design: 1, *BRCA1/2* carriers diagnosed with breast cancer treated with BCT or mastectomy. 2, *BRCA1/2* carriers versus sporadic cancer diagnosis in women <42 years of age undergoing BCT. 3, *BRCA1/2*-associated cancer matched with sporadic controls for age and year of surgery treated with BCT. 4, *BRCA1/2*-associated cancer matched with sporadic controls treated with BCT. 5, *BRCA1/2*-associated cancer versus patients with family history of breast or ovarian cancer versus sporadic controls matched for age and year of diagnosis treated with BCT. 6, breast cancer patients with versus without a family history of *BRCA1/2* mutation versus sporadic controls matched for age and year of diagnosis treated with BCT or mastectomy. 7, Ashkenazi Jewish women with versus without the *BRCA1/2* founder mutation undergoing BCT. 8, Ashkenazi Jewish women with versus without the *BRCA1/2* founder mutation undergoing BCT.

*Reached significance on univariate analysis but was lost on multivariate analysis.

**This result was mitigated by and was no longer significant after the addition of chemotherapy.
